# From inclusion to independence – Training consumers to review research

**DOI:** 10.1186/1478-4505-6-3

**Published:** 2008-03-09

**Authors:** Carla Saunders, Afaf Girgis, Phyllis Butow, Sally Crossing, Andrew Penman

**Affiliations:** 1 School of Medicine and Public Health, The University of Newcastle, Wallsend 2287, NSW, Australia; 2 The Cancer Council NSW, Woolloomooloo 2011, NSW, Australia; 3 Centre for Health Research & Psycho-oncology (CHeRP), The Cancer Council NSW, University of Newcastle & Hunter Medical Research Institute, Newcastle 2287, NSW, Australia; 4 Medical Psychology Research Unit, University of Sydney, Camperdown 2006, NSW, Australia; 5 Cancer Voices NSW, Greenwich 2065, NSW, Australia

## Abstract

Health and medical research invariably impacts on the lives of everyday people. Organisations in the developed world are increasingly involving the public in health research projects, and research governance structures and processes. The form the involvement takes varies, as does the level of involvement, from individuals, to groups, to the wider community. Lay community members can be trained to independently review health and medical research, and wider societal involvement in funding decisions, can be effectively fostered. The theoretical foundation, design and development of a task based consumer-training program, including a number of enabling factors to support the success of such training are presented. This work is likely to be of value to those planning to train consumers in technical or complex areas.

## Background

Purposeful public involvement in health and medical research has been an emerging agenda item in research governance processes in many economically developed nations for over a decade. Its importance arises, in part, out of an egalitarian desire to improve public access to and understanding of science [1], which was a key recommendation in a 1999 strategic review of Australian research and development [2]. Reported benefits of consumer involvement include raising the awareness of scientists to the realities faced by community members with particular health problems, a greater acceptance and uptake of research findings in the community, and more efficient use of research resources [3,4].

Peer review of research funding applications involves an assessment of scientific merit. Each scientific reviewer is assumed to have the expertise to judge the feasibility and suitability of proposed research methods and analysis, and the capacity and track record of the research team. Whilst consumers are not involved in the assessment of the scientific value of research applications, they are included on peer review panels by an increasing number of organisations around the world to provide lay input concerning the public significance of the research [5-10]. Involving consumers in research review requires developing realistic expectations of what they can and cannot contribute to an existing review process. Clear expectations will, in turn, increase the likelihood of appropriate opportunities being identified for effective and meaningful consumer input and ensures that the right balance between expert and lay assessment of research is struck.

The Cancer Council NSW [11] (CCNSW) is a large publicly funded charity that supports a significant proportion of Australia's cancer research. Cancer Voices NSW [12] (CVN) is the independent, peak advocacy organisation for all people affected by cancer in New South Wales, Australia. Striking a balance between funding research that has significant scientific merit and research that responds to the priorities of the community that finances it, has been a long-standing goal of the CCNSW and CVN. An analysis of the CCNSW research funding structures and procedures identified an avenue for the independent assessment of research grants by consumers. A recently developed national framework for community participation in Australian health and medical research guided the broad strategy components [13]. To ensure impartiality in the assessment process, the CCNSW developed a consumer research appraisal tool that is underpinned by the findings of purpose designed qualitative research [14]. Each criterion within the appraisal tool reflects prevailing community values relevant to making health research funding decisions. These include the level of positive impact on human lives, whether the research can be applied in the real world, the extent to which it will be available to all who could benefit by it, how soon the research was likely to be available in clinical practice, and whether consumers were involved [14]. The availability of this tool provides the opportunity for consumers to independently and objectively judge research applications, subsequent to scientific review, for the CCNSW.

As the specialist nature of research review is significantly different from most other activities of consumer representatives, detailed, quality training is essential. Increasing public interest in health and medical research, along with policy directives for improving public participation in research, have created a global opportunity and market for consumer education programs and has led to a recent growth in the number and type of such training courses [15-18]. However, there have been no information or published papers detailing the training of consumers to independently judge cancer research funding proposals and further research to develop different training methods and education for consumers has been recommended [19].

The purpose of this paper is to report on our experience in developing a task-based training program to support an independent consumer review of research grant applications. This paper summarises the theoretical framework for the development of this training, the structure of the course, the lived experiences of the participants, and a range of important enabling factors to effectively support consumers in research review. While this work is likely to help those who wish to train consumers in an independent research review process, it may also be valuable to others who intend to train consumers in technical or complex areas where there are few, if any, existing resources to inform effective development and successful implementation.

## The Training

### Design

The training program was first conceptualised and implemented as a pilot initiative with an expectation that it would proceed to a more systemic ongoing program. Whilst we could find no established instructional design principles or standardised development approaches specifically for training consumers to review research, the complex yet practical nature of research review lent itself to a task based instructional approach. According to the 'situated cognition' movement, which takes from Vygotskys' activity theory [20-22] and more recently from developmental work in educational psychology [23], learning is maximised if the context for learning resembles the actual situation in which the instruction will be used. Task based learning is a model that organises learning around an activity [24-26]. Central to the process is that the task, for which training is developed, is applied in the real life context. The key principles of the model are that instruction should reflect the complexity of the thinking and work that learners are expected to be able to do in the real world circumstance, and that learners must engage in active practice to establish genuine connections to the real situation [27].

To ensure the training involved adequate information and relevant practice of the required task, we first identified all the training elements in a storyboard format. This provided a reference point by which the design process could follow. Three aspects of learning (awareness, knowledge and skill) and three formats of delivery (oral information, interaction and written resources) were subsequently identified. The development of a matrix, combining the learning and delivery areas, provided a simple means of representing and organising the content and linkages of the training elements (Table 1).

**Table 1:** Consumer Research Review Training Matrix

	**Awareness **(Need to be aware)	**Knowledge **(Need to act in accordance)	**Skill **(Need to apply new knowledge)
**Oral Information **(Fundamental to research and research review. *Verbally communicated during the training*)	* Orientation to the organisation, including a discussion of its mission and philosophy * Broad explanation of research * Current research funding processes * Existing research review mechanisms (scientific merit review, ethical review) * Spectrum of cancer control research (range of possible investigations and core research disciplines)	* Purpose and rationale for consumer review * Role and responsibilities of consumer review panel members * Role and responsibilities of the chair of the consumer review panel * Consumer review principles (conflicts of interest, confidentiality, openness, efficiency) Ground rules (need for participants * to consider their own and others principles and biases etc) * A spirit of cooperation, teamwork and mutual respect needs to prevail during panel discussions.	* Each application is scored independently and not in comparison to other applications under consideration * Reviewer may abstain if they do not feel comfortable about providing a score * All participants can offer their perspective of each proposal * The final grade for each proposal should take into account the nature and intent of the research and the relative importance of each consumer review criterion.

**Interaction **(Necessary exchange of information, ideas, and opinions)	* Participants to ask for clarification if they are uncertain about an issue that may arise during the workshop * Question time allocated into each presentation * Verbal feedback on the effectiveness and clarity of the training sought throughout the training * Participants to be asked to continue to feedback even after they complete the training * If valuable group discussions occur during planned presentations, dedicated time to be set aside to discuss and clarify the issue	* Participants to write questions or concerns down so they can be dealt with during the workshop * Participants required to complete written evaluation forms and pre and post training surveys * Prior to the panel meeting, members to inform the Chair or support personnel of any issues and/or concerns they may have about the research applications or taking part in the review process. * Participants to compile a list of any unfamiliar concepts, terminology etc during the review process * Perceptions of lack of skill or knowledge are discussed in an open forum and addressed through reference (and necessary changes) to the formal terms of reference	* The nominated key spokesperson (each participant assigned 2–3 applications to review in detail) to describe the proposed work and give an assessment of its strengths and weaknesses (against the consumer review criteria) * The chairperson (group elected) to summarise the full review panel's discussion and asks the panel members whether their recommended scores remain the same or are changed following the discussion. * All panel members to verbally agree on the final ranked list of the proposals. * The chairperson to compile a summary report which includes the average of the individual reviewer's scores and a summary of the panel's discussion of each of the proposals and the priority listing of proposals

**Written Resources **(Enhance and/or reinforce learning)	* List of research related abbreviations and acronyms * Glossary of research and other relevant terms * General information on cancer and research * Fact sheets on each cancer research discipline * Relevant examples of research in each discipline * Flow diagram of research funding and review processes * Frequently asked questions (FAQs) on scientific merit and ethical review	* Formal terms of reference for the review panel * Detailed list and explanation of the responsibilities and ground rules for the review process * Consumer review principles fact sheet * FAQs on conflicts of interest and confidentiality	* Step by step guide to the consumer review of research applications * Review criteria guidelines * Reviewer forms and scoring ranges * Tips on reviewing research applications and trouble shooting guide * Contact details of chairperson and support personnel, travel directions and map to the CCNSW

In designing the training it was important to make sure that participants were not taken out of the process. As the role of reviewer is a social activity, it was felt that participants would benefit greatly from activities that led to group interaction and active participation in the training process itself. Critical thinking skills that challenged consumers to continuously seek new ways of understanding the information before them and to recognise the complexities within research funding submissions were considered especially important. The design also focused on developing the necessary interpersonal requirements such as working as part of a team, the ability to negotiate and understand group dynamics and conciliate to assist the resolution of complex issues. The ultimate requirements of the training concern the development of appropriate cognitive and practical skills so each consumer review panel member becomes competent to conduct an assessment of research using predetermined consumer review criteria.

Training applicants were purposefully selected after a general recruitment process via newspaper advertisements and consumer health organisation newsletters. Inclusion in the training was based on a number of aspects, including whether they had an interest in cancer research, a willingness to become familiar with research terminology, disciplines and concepts, experience in consumer centred cancer groups and organisations, and the ability to keep up to date with current consumer issues via consumer networks and associations. Screening and interviewing potential training participants minimised the effect of real-world factors such as differing levels of experience among potential members and facilitated their commitment to, and expectations of the training. It showed potential participants that the CCNSW took both the training and their time seriously. Screening also gave the opportunity to match personal characteristics and skills with the needs of the review task such as the ability to consider the views of others. Those subsequently trained were a mix of cancer survivors and members of the general public affected by cancer through the experience of family members and/or friends.

Cooperation and collaboration among experts was also crucial in the development of the training program. An advisory committee, which included informed consumer representatives, was established to advise the development of necessary training materials and methods.

The design of the training program included the development of detailed written guidelines (Additional file 1) to support the review process and explain the review criteria in detail. Examples of research summaries in lay language were made available in the guidelines to further support the process.

When dealing with relatively complex information it is important that learners are able to access detailed information in a number of forms. A comprehensive manual was provided to each participant with information covered in the training and additional resources that were felt could assist the learning process, such as a step-by-step written guide of the consumer review process (Additional file 2).

Training programs are in many cases not developed and managed as a product that is able to be re-used and adapted by others. A broad approach to facilitate the replicability of the training program was adopted. Table 2 provides the outline of the final training program.

**Table 2:** Outline of the Cancer Council NSW Consumer Review of Research Training Program

**Day 1**	**Day 2**
**Introductions and Welcome**	**Welcome to day 2**

**Setting the Scene**	**About the Consumer Review Panel *(the panel of trained consumers who will review and prioritise CCNSW research funding applications against established criteria)***
• About the Cancer Council NSW	• Purpose of the consumer panel
• Cancer research in Australia	• Role of panel members
• Background to consumer involvement in research at the Cancer Council NSW	• Role of the Chairperson
	• Operating guidelines and protocols for the panel
	• *Questions/views/training improvement ideas*

**Overview of Training **– expected outcomes, ground rules, evaluation.	**Research review**
	• Consumer Review Criteria (development and use)
	• *Questions/views/training improvement ideas*

**Definition and Scope of Health and Medical Research **(for the purposes of the training)	

**Current Research Funding and Review Processes**	**Testing the System (practicing research review)**
• National Health and Medical Research Council	• Review example research funding applications
• Cancer Council NSW (including types of research funded by CCNSW)	• Individually apply the consumer review criteria *(identified from recent research on important aspects the NSW public feel are important when judging the value of research) *to each application
• Definition and importance of ethical approval and scientific merit review	• General discussion and debate
• *Questions/views/training improvement ideas*	• Determine a (panel agreed) ranked order of applications for funding
	• Debrief on the process, experience, problems encountered etc
	• *Questions/views/training improvement ideas throughout the practice session*

**Overview of Research Disciplines **(and importance of each for cancer control)	**Next steps and other research involvement opportunities **(with the Cancer Council NSW)

**Basic Research**	**Summary of day 2 ***Questions/views/training improvement ideas*
**Epidemiological Research**	
**Clinical Research**	
**Behavioural and Psychosocial Research**	
**Cancer Screening Research**	
**Health Services Research**	
**Summary of day 1 ***Questions/views/training improvement ideas*	

### Delivery

To cover all the necessary information and skills required by research reviewers, the training mixed didactic presentations on each cancer research discipline, with group and practical sessions in actual research review using previously funded grant applications and suitable lay summaries. Complex information was considered best taught as discrete elements so a lay overview of each research discipline was designed and presented separately by prominent experts in each of the fields. Written lay summaries of each research discipline were developed by CCNSW staff and edited and approved by each relevant expert (Additional files 3 and 4).

The positive value of involving specialist researchers to describe their disciplines without compelling trainees toward any particular type of research over another was a recurring finding throughout all elements of the evaluation of the training. Other important contributors to increasing participant knowledge and skill was learning from each other in group discussions, and from staff who routinely answered questions, clarified meaning and provided any additional information that was required.

It was recognised that an atmosphere of respect, trust, useful dialogue and effective practice was necessary if we were to successfully train consumers to review research applications in two days. In the practice review session participants were supported to apply the review guidelines and assist us to work out the finer details of the review process. Use of actual research proposals allowed us to efficiently obtain a snapshot of review skills and participants' baseline confidence and communication techniques. This fostered a productive discussion during the feedback sessions, which focused on how to more effectively undertake the review process.

Participant input was reviewed regularly throughout the two-day program. The ongoing critical reflection of the training led to several modifications such as allowing more time for several of the sessions, improvements to the type and sequencing of information available in the training manuals and the need for greater interaction with speakers. Participants were given the opportunity to describe what they had gained from the practical experience:

"Increased my confidence tremendously."

"I was surprised that I could 'get it'."

"Doing the review made the information so much more meaningful."

### Application

The victory lap cannot realistically be taken until the training actually produces the results that were intended. The ability to move from training to practice is dependent on the relevance of the training to the particular task. The reliable measure used to determine the effectiveness of the research review training was an assessment of the process and outcomes of consumer review. The assessment of the first round of consumer review of research found that all participants, while having a range of experiences and abilities, were able to strictly apply the consumer review criteria to research proposals which, as the training intended, significantly minimised subjective input. It also found, that the application of the criteria limited unnecessary dialogue which made the review process efficient and professional. Most importantly, the participants ranked higher in priority order those proposals that best met the requirements of the consumer review criteria.

In making final funding decisions, an oversight committee allocates equal weighting to applications prioritised secondarily through the consumer review process and those prioritised by scientific merit review alone. Significant discordance between consumer and scientific review is resolved in open discussion by a purpose-convened group that includes both trained consumers and researchers. The success of the CCNSW consumer review of research, which is now an enduring research governance process, has led to its keen adoption by other organisations [28].

It is possible to train consumers in research review and achieve the desired effect. A summary of important enabling factors to effectively support consumers in research review is provided in Table 3. However, since this reform is a challenging concept and a major departure from the old strategies, proper orientation of all stakeholders on the new approach is critical to its effective application. Researchers submitting proposals for funding by the CCNSW and those involved in the final selection process were made aware of the changes and implications of the consumer review process through written protocols and guidelines, and formal discussions including updates and refinements in the process. It is essential that appropriate organisational information be constructed in parallel to training so the newly developed reform can become truly embedded.

**Table 3:** Enabling factors to support the independent consumer review of research

**Themes**	**Enabling factors**
**Participants**	Establish whether future participants have a true interest in the training and research review process.
	Identify and work to the circumstances and capacity of the participants *e.g. where possible align key spokesperson status for each research funding applications with the experience and knowledge of individual consumers*.
	Include participant views and requests in the development of the training and review process by calling for regular targeted feedback.
**Systems**	Provide clear formal policies and protocols for the review process and related responsibilities, including conflict management guidelines.
	Make available dedicated resources *(costs associated with training manual and program development and production) *and visible staff support that continue to grow with the needs and capabilities of the participants.
	In parallel to the training, ensure that appropriate organisational information *(policy and procedure in relation to the consumer review structure and process) *is constructed in line with all requirements so the newly developed course of action is workable and becomes ingrained.
**Instruction**	Ensure there are adequate confidence gaining opportunities (such as trialing an actual review process) to increase familiarity and experience.
	Ensure the end outcome (research review) is the focus of all aspects of the training.
	Provide appropriate information targeted specifically to the research review process through relevant content filtering to enable continual update of resources needed by, or useful to, participants (including information separated out and identified by participants).
	Involve confident experts in the design and delivery of the training and review process.
**Motivation**	Establish a genuine perception among participants that they are making a difference.
	Create a sense of recognition and ownership of the review task.
	Generate and maintain enthusiasm so that the distinct focus and vision can be achieved.
	Promote group cohesion and unity, and ensure the 'culture' developed is a positive one.
	Allow free speech and the free flow of information among participants.

## Conclusion

Consumer training in research review underpins an important change in CCNSW research governance [29]. The new research review process encompassing consumer review, subsequent to scientific merit review, is designed to offer the highest promise of fulfilling community needs. It provides an approach to independently assess the social value of research through the use of empirically identified criteria considered important by community members. The first year of the consumer review process led to the funding of three out of twelve research funding applications that would not have been previously considered, but which better served the needs of the community.

We endeavoured to create a training program that would meet the needs of the consumer reviewers and would be sustainable in the long-term through a dedicated, yearly training program that is offered to existing trainees and new recruits.

The CCNSW and Cancer Voices NSW have achieved their goal to ensure that a trained group of consumers is available to participate in grant review. Consumers can reliably and effectively undertake a challenging and complex requirement if they are given training that is aligned to their ability and specific to the task. The involvement of expert scientists is critical to the continued value of this program and to make sure participants benefit from the wealth of experience that these individuals have to offer. As national and international policy directives continue to build on the momentum that presently drives public participation in research, careful attention must be paid to both the organisation and quality of essential training.

## Authors' contributions

CS participated in the design, coordination, delivery, evaluation and ongoing improvements to the training. AG participated in the design and delivery of the training. PB participated in the design and delivery of the training. SC participated in the design, coordination and delivery of the training. AP participated in the design and delivery of the training. All authors read and approved the final manuscript.

## Supplementary Material

Additional file 1 Consumer Review Guidelines. A resource to support the review process and explain the review criteria in detail. Click here for file

Additional file 2 Consumer Review Process. A step-by-step written guide of the consumer review process. Click here for file

Additional file 3 Basic Research. A lay fact sheet explaining the basic (laboratory) cancer research discipline. Click here for file

Additional file 4 Behavioural Research. A lay fact sheet explaining the behavioural cancer research discipline. Click here for file
