# Supplementary material for: From inclusion to independence – Training consumers to review research
Source: Health Res Policy Syst. 2008 Mar 9;6:3. doi: 10.1186/1478-4505-6-3 (PMC2292183; doi:10.1186/1478-4505-6-3)
Supplement: Additional file 1 — Consumer Review Guidelines. A resource to support the review process and explain the review criteria in detail. [file 1478-4505-6-3-S1.pdf]

<u>Additional file 1</u> Consumer Review Guidelines

CONSUMER REVIEW OF RESEARCH GRANTS 2008

Guidelines for the preparation and review of research grant applications

The Cancer Council New South Wales

Introduction

It is important to The Cancer Council NSW that the research it funds is valued by researchers and the community it serves. A growing need to promote stronger relationships between researchers and the general public was identified by Cancer Voices NSW (the peak NSW consumer advocacy organisation for those affected by cancer), with the specific aim of involving consumers in the decision-making processes behind research funding.

The Cancer Council NSW, in consultation with Cancer Voices NSW, has established a formal process to allow consumers to have a say in funding external research. The Consumer Involvement in Research Project resulted in the development of specific consumer review criteria and the training of a consumer panel in the assessment of research applications on behalf of cancer consumers and the wider community.

The criteria are designed to assist members of the Consumer Review Panel to judge research proposals for funding. The objective of these guidelines is to ensure quality and consistency in the use of consumer review criteria and provide guidance to researchers to assist them in developing research proposals that meet the requirements of The Cancer Council NSW.

A description and explanatory notes for each of the criteria is provided below. Questions that the Consumer Review Panel is asked to consider when making scoring judgements are also provided. An example response, which rated highly by the Consumer Review Panel, is provided for each criterion.

All applicants seeking research grant from funding The Cancer Council NSW must complete a Consumer Review Form addressing the consumer review criteria.

1. Extent of benefit

Will the findings potentially have an important positive impact on human lives, including any of the following aspects: disease causation, prevention, diagnosis; treatment; physical and/or mental and/or social wellbeing; quality of life, dignity, survival?

Description

This criterion provides the opportunity for the applicant to explain the potential for the proposed research to have a direct, beneficial impact on the lives of people affected by cancer. To adequately address this criterion, researchers should provide a detailed lay summary of their research. Consumers are not expected to have a science or research background, but have received preliminary training in research design and process.

It is important to consider benefits from the perspectives of the general public as well as those more directly affected by cancer. Some examples are:

* Identifying the mechanisms by which cancers arise.
* Developing ways of individualising cancer treatments.
* Identifying and/or testing effective ways of preventing and/or treating disease. This might include improvements in the environment or individual behaviours.
* Identifying those at high risk of developing cancer.
* Improving existing or identifying new cancer care delivery approaches, treatments and/or diagnostic methods.
* Improving access to information, and the quality of information available.
* Easing physical and/or mental suffering of those affected by cancer.
* Maintaining or rebuilding dignity and quality of life.

* When assessing this criterion, the Consumer Review Panel may consider some or all of the following:

* Has the researcher explained how the research will generate tangible benefit/s to human life? Are there a number of benefits?
* Has the researcher indicated the probability, magnitude, and/or duration of these potential benefits?
* How important do you think the benefit(s) is?

Example:
Most cancer cells harbour chromosomal defects, which affect gene structure, number and function. The development of multiple chromosomal defects in individual cancer cells is implicated in the progression of premalignant conditions to aggressive malignancies. Our investigations will delineate the molecular processes that are responsible for the development of chromosomal abnormalities during the development of cancer and will define the role of these chromosomal alterations in the multi-step process of cancer development. This information will enable the development of new strategies for the treatment of a broad spectrum of malignancies that are characterised by multiple chromosomal changes and unstable genomes. These include some of the most common types of human cancers, including breast, lung, prostate and colon cancers, as well as some childhood malignancies. Treatment of cancers with multiple and/or evolving chromosomal abnormalities is extremely challenging, as constantly changing genetic material presents a very complex target. Hence the treatment of cancers with evolving chromosomal abnormalities usually involves the use of chemotherapeutic agents that are broadly toxic to cancer cells and normal tissue, which seriously impacts on quality of life during treatment. The elucidation of pathways that contribute to the evolution of gross chromosomal changes will enable the development of novel therapeutics designed to specifically impede these changes. Intervention such as this will prevent the progression of pre-malignant conditions to more aggressive cancers, thereby circumventing the requirement for more toxic treatment and improving quality of life and the chances of survival. These investigations will also define molecular markers that may be used to identify patients at risk of progression, which will enable treatment to be tailored to the prognosis and thereby deliver optimal outcomes.

2. Pathway for realising the benefit

Is there a clear description of the steps required to reach the stated end benefits of the research?

Description
Consumers recognise that further steps are often required for the benefit of research to be realised. These steps might include additional laboratory based research, testing on humans, changes in clinical practice, product development, regulation/law and/or policy changes. For each step to realising the benefit, there are likely to be related investigations, costs and risks.
Consumers also recognise that outcomes achieved in a single body of work may make significant advances to the knowledge of cancer, but not reach a point where a final benefit, directly applicable to humans is achieved. Identifying the pathway required to reach an applicable benefit, and highlighting which steps the proposed research will be addressing, will allow the reviewer to judge when and how the results of the proposed research project will be realised.
When assessing this criterion, the Consumer Review Panel may consider some or all of the following:

* Has the researcher provided a brief description of the broad steps or stages required to reach the stated benefits of the research?
* Do the steps or stages appear reasonable?
* Are the steps or stages achievable?
* Are there any significant gaps in the steps or stages required to reach the stated benefits?
* Do the steps or stages represent significant constraints to achieving the actual benefits of the research?

Example:
We are working at the edge of human knowledge of the causes of breast cancer, seeking to discover the mechanisms that govern the normal development and function of the breast, as these mechanisms are hijacked during carcinogenesis. Our knowledge of these mechanisms is primitive at best. Our currently effective drugs in breast cancer were developed to target some of the discovered mechanisms that operate in normal mammary development. Without an effort to discover and understand new mechanisms there can be no new cures for breast cancer.
The path from new knowledge to therapeutic is long and many promising lead compounds fail on the way. The development of Tamoxifen, Herceptin and Iressa demonstrate that the development of therapies against molecular targets with normal developmental roles can be achieved with spectacular results. The basic steps in this pathway are:

1. *Identify a target molecule (task complete: Elf5)*
2. *Demonstrate the target is involved in cancer (purpose of this application)*
3. *Investigate the mechanism of target action*
4. *Devise a strategy for a small molecule to modulate the activity of the target using the mechanism defined in 3.*
5. *Test the small molecule in disease models (cell/animals)*
6. *Formulation and pharmacologytoxicology testing*
7. *Phase1 human clinical trial*
8. *Phase2 human clinical trial*
9. *Phase3 human clinical trial*
10. *Phase4 clinical trial and market drug.*
11. *Withdraw drug if serious adverse events occur in large population following release.*

3. Potential for application of findings

Is there potential for real world application of the findings in the long-term?

Description
While research may have the potential to lead to human benefit, it is important to consider whether or not the benefit can actually be realised in the real world. There will be times when real world hurdles such as resources, technical challenges, public and/or professional acceptability, availability, risks/adverse consequences, process, policy and/or legal barriers and other constraints will affect whether or not the research findings will be put into practice and/or made available to the public.

When assessing this criterion, the Consumer Review Panel may consider some or all of the following:

* Is it likely that the findings of the research will be able to be put into practice (in either the short, medium or long term)?
* Are there likely to be significant barriers to putting the research findings into practice?
* How compatible are the research findings likely to be with existing laws, public policy, resources etc?
* How will the research findings affect current ways of working (eg clinical and other practices)?
* Where relevant, does the researcher include the groups they will work with to overcome barriers to applying the findings of this research?

Example:
Raising pathogen-specific T cells in vitro (in our case against CMV) has already been shown to be a simple and economically viable procedure in the laboratory setting. This trial aims to test the safety and efficacy of these cells as a preventative treatment for CMV reactivation and, if successful will be adopted as a standard treatment for all transplant recipients. Not only does this procedure have a current application at the clinical level, the principle (of raising disease-specific T cells) may be applied to other pathogens that threaten transplant recipient health.

4. Equity

Is there adequate justification for the selection of the study sample that demonstrates potential for equity, eg the research does not exclude groups who could potentially benefit from its outcomes, **and/or** it addresses an under-studied group **and/or** a group with a high burden of illness?

Description
Equity in research asks the question 'who benefits?' Equity in research is commonly thought of as striving for equal benefit from research. There is no universally accepted best or right answer for how research benefits should be distributed in society, although ideally everyone who could have an opportunity to benefit from research should.
For example, a research project that focuses on a particular cancer or group of people, should explain the rationale behind this focus, and address how the benefits of the research may be expanded to other groups in the future.

When assessing this criterion, the Consumer Review Panel may consider some of the following (the research is not required to meet all these expectations)**:**

* Does the researcher explain how the findings could be generalised or applied to similar people outside the research?
* Does the research have the potential to provide benefit across all relevant persons, groups and/or places?
* Does the research address an under-studied group?
* Does the research address a group with a considerable burden of illness?

Example:
The type of bowel cancer that is caused by wrongful silencing of the MLH1 cancer-protector gene by methylation is most frequent in women aged above 60 years. Therefore our study will logically focus on this group, since it has the highest burden of disease. Within this group there will be equity irrespective of factors such as race and socioeconomic status.

5. Consumer involvement

Have consumers been involved in the planning, implementation and/or information dissemination stages of research?

Description
Whilst scientific merit may be sufficient to identify research of high quality and the greatest potential for success, it does not take into account the needs of consumers and the general community. At present, consumers have little input into the decisions behind what research gets funded, and there is a growing desire to make publicly funded research more responsive to public needs.
There are many opportunities for consumer involvement in all stages and all types of research. A reasonable and appropriate level of consumer involvement may vary, depending on the nature of the research being undertaken, but could include almost any kind of interaction between consumers and researchers. There is no single best method of involvement. Some examples are:

* Work with researchers on defining or refining the research topic and developing a research proposal
* Be included in project/institution advisory committees
* Conduct lay reviews of research proposals.
* Support the development of lay summaries.
* Assist researchers to pilot a research questionnaire.
* Produce newsletters for members of their organisation that chart the progress of research.

While there is a need to build the capacity of consumers to become involved in research, there is a parallel need for researchers to learn how to effectively involve consumers. One effective avenue for public involvement is via informed consumer representatives.

a) Development phase

Have relevant informed consumers (eg from consumer or cancer groups such as Cancer Voices NSW, Breast Cancer Action Group NSW, Consumers Health Forum, cancer support groups etc) been involved during the development of the research proposal? When assessing this criterion, the Consumer Review Panel may consider some or all of the following:

* Is consumer involvement described?
* Have researchers identified the preferred approach of consumers for involvement in the research?
* Are there formal processes/structures in place that link the researchers with consumers?
* Considering the nature of the research, is the extent and type/s of consumer involvement appropriate?
* Are there plans for consumers to be involved in the dissemination of research results?

Example:
Consumers have been instrumental in SNAC1's success. Our Trial Management Committee has included 2 consumer representatives since its inception: Avis McPhee (Breast Cancer Network Australia) and Leonie Young (Consumer Advisory Panel, ANZ Breast Cancer Trials Group). They helped develop and review the: protocol, patient-information, quality of life instruments, newsletters, and presentations to researchers, consumers and the press. The contributions of their organisations and the NBCC, NBCF, and Cancer Councils have also been vital.
Our consumer representatives were instrumental in discussions and decisions to establish SNAC2 and both have accepted invitations to its Trial Management Committee.

b) Ongoing involvement

Is there a plan for ongoing consumer involvement in the research?
When assessing this criterion, the Consumer Review Panel may consider some or all of the following:

* Is ongoing consumer involvement described?
* Considering the nature of the research, is the extent and type/s of ongoing consumer involvement appropriate?

Example:
Ongoing consumer involvement will be promoted by presentations of the results of our research at regular Garvan public seminars held at the institute and NSW regional centres. These feature researcher, clinician and patient perspectives in lay language and since two-way dialogue is actively encouraged, will enable consumer feedback.

6. Dissemination of results

What is the plan for circulating lay information about **all** results of the research to other researchers, human participants and the general community?

Description
It is in the interest of research participants, media, consumers and the wider public that research findings generated by publicly funded research are made accessible in a context, format and language that is appropriate for the relevant lay audience. It has been found that the main barriers to an improved public understanding of health and medical research are the lack of public education and information, and the perceived misrepresentation effect of mass media communications.

When assessing this criterion, the Consumer Review Panel may consider some or all of the following:

* Does the researcher/s explain how and when they will give human participants access to research results, including details of any intervention they were exposed to during the research?

* Does the researcher/s explain how they will give other researchers, participants and the general community access to research results?
* Will research findings be available to consumers and the media in formats, concepts and language they can easily understand?
* Are there valid reasons for the researchers not to communicate the results of the research?

Example:
Results will be disseminated in a range of formats depending on the target audience:
Other researchers: Seminars, conferences and published literature.
Human participants: AOCS Newsletter and presentations to the Gynaecological Cancer Patient Support Group at Westmead Hospital.
The general community: Presentations to groups such as those given in the past including the Holroyd Rotary Club, the NSW Country Women's Association and the Cancer Council NSW Donors Luncheon; visits to the lab by community groups, patients and their families organised by the Westmead Millennium Foundation; Annual Reports available on our institutional website and related websites including those of funding bodies such as the Cancer Council; and, if warranted, may include the lay press and TV news once results have been peer-reviewed and published in scientific journals.
