# Supplementary material for: From inclusion to independence – Training consumers to review research
Source: Health Res Policy Syst. 2008 Mar 9;6:3. doi: 10.1186/1478-4505-6-3 (PMC2292183; doi:10.1186/1478-4505-6-3)
Supplement: Additional file 2 — Consumer Review Process. A step-by-step written guide of the consumer review process. [file 1478-4505-6-3-S2.pdf]

**Additional file 2**

## Consumer Review Process

---

### Review Process

- Two to three weeks prior to the review meeting, panel members will be forwarded:
  - The original research proposals
  - A 1-2 page lay summary of each proposal
  - Notification of which proposal/s they are to be key spokesperson for (this will often be based on experiences/ knowledge/preferences)
  - A grading scale
  - A conflict of interest form
- Members will be asked to identify in writing any conflicts they have with each of the proposals.
- Members must assess each research proposal as it is presented, and are not permitted to alter the proposals in any way.
- Panel members will address the appropriate review criteria and indicate the score they will initially assign to the proposal.
- If a panel member does not feel comfortable about providing a score, they may abstain.
- Each proposal is scored independently and not in comparison to other proposals under consideration.
- Prior to the panel meeting, members should inform the Chair or CCNSW support personnel of any issues and/or concerns they may have.
- Panel members will meet at the Cancer Council head office to discuss and give a ranking to each of the research proposals.

**It is important that a spirit of cooperation, teamwork and mutual respect prevail during panel discussions**

- In the event of a panel member withdrawing due to illness or other issues, the CCNSW will replace the member with another consumer with similar and appropriate expertise (research trained).
- At the commencement of panel deliberations, the chairperson asks the panel members if there are any undeclared conflicts of interest that may influence judgements and checks all conflict of interest forms (refer to the *Consumer Review Principles* section for information on conflict of interest).
- The chairperson then calls on the key spokesperson for the first proposal to provide the group with a description of the proposal.
- Each reviewer will then have an opportunity to:
  - Describe the proposed work and their assessment of its strengths and weaknesses
  - Offer their perspective concerning the proposal
  - Ask the cancer council support personnel to clarify any unfamiliar concepts, terminology etc
- The chairperson summarises the full review panel's discussion and asks the review panel members whether their recommended merit scores remain the same or need to be changed following the discussion. Each panel member will be given an opportunity to change the scoring against each criterion after hearing the perspectives of others.
- Each person gives their final scores for each proposal to the chair who adds them and then divides them by the number of all participating members in the group.
- A spokesperson description and full panel discussion of each proposal will continue in turn until all the proposals have been considered and have been assigned an agreed grade (a grading scale will be provided to help rank proposals).
- All panel members should agree on the final ranked prioritised list of all proposals.
- The chairperson compiles a summary report which includes:
  - The average of the individual reviewer's scores
  - A summary of the panel's discussion for each of the proposals
  - The priority listing of proposals
