# Supplementary material for: From inclusion to independence – Training consumers to review research
Source: Health Res Policy Syst. 2008 Mar 9;6:3. doi: 10.1186/1478-4505-6-3 (PMC2292183; doi:10.1186/1478-4505-6-3)
Supplement: Additional file 3 — Basic Research. A lay fact sheet explaining the basic (laboratory) cancer research discipline. [file 1478-4505-6-3-S3.pdf]

**Additional file 3**

The Cancer Council
New South Wales

# Basic Research

---

**What is basic research?**
Basic research seeks to understand the biology of cancer and the fundamental mechanisms by which cancers arise. Basic research provides us knowledge of the intricate mechanisms that sustain life and the errors in the function of living systems that are responsible for disease. Such as understanding the role of gene mutations in producing changes in cell function, how normal cell function changes in cancer cells and the discovery and development of new anticancer drugs or other anticancer therapies.

**Why should we do basic research?**
With some diseases not enough is known to begin a treatment that might be successful. Often there aren't adequate models for the study of a disease, or so little is known that the design of experiments is impossible. Furthermore, in order to tackle major diseases of today such as cancer, heart disease, arthritis, and diabetes, we need a broader base of knowledge. We need to know more about the specific cellular and molecular changes involved in the development of these conditions. By providing this knowledge, basic biomedical research forms the foundation for advances in the diagnosis, treatment, and prevention of such diseases. Basic research may appear to be only indirectly related to a specific disease, but the same basic research finding is often relevant to many diseases.

**What are the common types of research questions answered by basic research?**
Basic biomedical research can provide answers to:

1. The primary biologic nature of cancer; cancer genetics; how tumours form, progress, and metastasize; and how they can be prevented and treated.
2. The unique role that viral agents, their components, or products may play in human cancer.
3. Angiogenesis - a process by which tumour cells develop the blood supply necessary for their ongoing growth and development. A large focus of cancer research is the development of a better understanding of this process to allow cancer therapies to be directed specifically at inhibiting angiogenesis.
4. Improving cancer diagnosis - a diagnostic tool coming directly from basic science laboratories is the magnetic resonance imaging (MRI) instrument. MRI uses magnetic and radio frequency energy to reveal new information about the chemistry of the living body. MRI was initially used only in research laboratories to study chemicals in test tubes. Now, MRI machines are being used in conjunction with computers to provide detailed pictures of the body's interior including exposing tumours and determining if they are malignant or benign.

**Examples**
Questions addressed by basic researchers might include:

- Which genes are most important in specific cancers?
- How do cancer-causing genes function in the cell to control cell growth, replication or death and to promote metastasis?
- How can the immune system be best armed and activated to protect against the development of cancer and to eliminate residual cancer cells remaining after chemotherapy or radiation therapy?
- How do stem cells function and can they be further exploited therapeutically?

**Limitations of basic research**
The limitations of basic research have been characterised in a number of ways, often in a combined manner: by reference to its ultimate purpose (research carried out with the sole aim of increasing scientific knowledge); its distance from application (research on the basic aspects of phenomena); or the time frame in which it is situated (research in a long-term

perspective). Put simply, the more "basic" the research, the less obvious the connection to practical applications.

**Cost**

The growing cost of basic research due in particular to the cost of the instruments, equipment and infrastructure needed, as well as the complexity of the subjects with which basic research is concerned. For example, understanding the function of proteins at the cellular and subcellular level in living systems is possible due to recent developments in optical and computational technology. Basic research technologies are evolving in complexity, expense and becoming increasingly specialised. The enormous discovery possibilities that new technologies provide are balanced by a requirement for substantial and ongoing expenditure. For example, microscopes used in the latest cell-based research, cost more than $1 million each. It is probably important to remember that basic research is a relatively inexpensive investment compared to the cost of health care. “If we can reduce cancer deaths in the next forty years by 20%, we would save $184 billion” *(Access Economics report published 2003, Australian Society of Medical Research)*.

**Basic research methods**

Some of the research methods used in basic research include:

1. **Cell and tissue culture** - Cell and tissue culture technology is being used to learn more about a wide variety of human diseases including genetic diseases.
2. **Animal models -** The use of animal models, especially mouse models, is often an important step in basic science research. By using an animal model, researchers increase their ability to isolate and study certain features which would be too complex to study or impossible to isolate in the original system. In many cases, animals are better suited for research purposes and can provide faster and more accurate results than humans. The shorter life spans of most animals make them better suited for studies where scientists must predict results in a given lifetime or over many generations. Rats, for example, go through their life cycles in about three years, compared to seventy years or more in humans. Conditions such as diet, light, and temperature can also be controlled more readily in animal studies, which increase the validity of experiment results. For some research, it's simply not ethical to experiment on humans. Most doctors, for instance, would not encourage their patients to take a drug for which the side effects are not yet known. Nor would they breed humans to get a pure genetic strain in order to perform a study.
3. **Computer modelling and image analysis -** Researchers use models because they help to answer questions that could not be answered using the original system with the technology and methods that exist. Medical image analysis has helped researchers in the development of treatments for a number of diseases, including breast cancer, colorectal cancer and heart disease.

**Who Conducts Basic Research?**

A variety of organisations and individuals conduct medical research. Basic research is mainly carried out by universities or dedicated research institutes that have the necessary equipment and infrastructure. Within these organisations, a wide array of individuals, both scientists and non-scientists, each with different educational backgrounds and talents, form a framework, which makes this research possible.
