# Supplementary material for: From inclusion to independence – Training consumers to review research
Source: Health Res Policy Syst. 2008 Mar 9;6:3. doi: 10.1186/1478-4505-6-3 (PMC2292183; doi:10.1186/1478-4505-6-3)
Supplement: Additional file 4 — Behavioural Research. A lay fact sheet explaining the behavioural cancer research discipline. [file 1478-4505-6-3-S4.pdf]

Additional file 4

Behavioural Research

What is behavioural research?

Behavioural research looks at how our behaviour can affect our chances of getting cancer. It tries to find out why people behave the way they do, why they sometimes behave in unhealthy ways and what motivates them to adopt more healthy behaviours.

Epidemiologists have identified a number of important behavioural risk factors for cancer, including smoking, sedentary lifestyle, and obesity. Behavioural research scientists can help to understand the determinants of these behaviours by investigating questions such as: Why do some smokers try to quit smoking while others do not? The aim is to identify important influences on such behaviours.

Findings from behavioural research can be put into practice at several levels, for example, at the individual (e.g. group nutrition, exercise, or smoking cessation programs), interpersonal (e.g. advice and support from one's physician for smoking cessation), organisational (e.g. healthy workplace policies), and societal level (e.g., mass media programs, tobacco control legislation, changes in school health education programs.

Why is there a need for behavioural research in cancer control?

It has been argued that the burden of illness associated with cancer could be significantly reduced if behavioural risk factors such as smoking and exposure to sunlight were reduced and screening for cancer of the bowel, breast and cervix increased. However, there is currently inadequate knowledge about how to achieve such changes. The most effective mechanism of identifying this is to carry out relevant, thorough behavioural science research in the area of cancer risk and cancer protection behaviours.

Research methods used in behavioural research

Research methods commonly used in behavioural research include:

- Broad measurement strategies for assessing the prevalence of target behaviours, including self-report, observational strategies, clinical judgement and biological or biochemical measures. Such findings from prevalence studies may highlight the need for educational and environmental interventions and suggest possible high-risk groups, and potential access points for implementing such interventions. This sort of information is crucial in ensuring cost-effective implementation of programs.
- Efficacy trials require an experimental design to evaluate whether interventions are able to produce change. Efficacy trials are important in order to demonstrate the value of behavioural strategies and ensure cost-effective allocation of resources. Results of such trials can be used to convince legislators and health policy makers about important policy changes that can potentially affect whole populations.

Examples of behavioural research:

Behavioural cancer research can include studies on lifestyle, physical activity, tobacco use, and palliative and end-of-life care. Examples include:

- Description and measurement of cancer related behaviours (eg. surveys of prevalence of smoking in children and adults; surveys of sun protection behaviours)
- Causes of cancer related behaviours (eg. studies of uptake of smoking and skin cancer prevention)
- Communication of cancer-related information, where research tries to identify source, message, channel and receiver factors that influence the effective and efficient transmission of cancer related information (eg. information-seeking patterns of cancer patients; audience responses to anti-smoking advertisements).

- Changing cancer related behaviours, where interventions to change cancer related behaviours are developed and evaluated (eg. interventions to encourage consumption of more fruit and vegetables).

Limitations
Self-report is one of the more frequently used methods for assessing the prevalence of target health behaviours because of its relatively low cost, and because it allows the estimation of the prevalence of target behaviours which cannot be easily assessed by other methods. However, there are several reasons for suspecting the accuracy of self-report. First, it relies on a memory of events sometimes going back over a number of years. Second, it presupposes both a knowledge of the behaviour (e.g. having a Pap test taken) and whether or not that behaviours was performed, when such a knowledge may not always be present. Third, given the degree of social pressure to conform to accepted health practice, and to engage in preventive health behaviours, some people may misinform in order to appear to be 'doing the right thing'.

Cost of behavioural research
The cost of behavioural research may extend beyond the costs of research staff salaries. Training and transport costs may also be required. Behavioural research often involves the active cooperation of research participants. Recruitment and involvement take time and money. As does the cost of telephone calls, postage, and other required resources.
